# Model-Centric or Data-Centric Approach? A Case Study on the Classification of Surface Defects in Steel Hot Rolling Using Convolutional Neural Networks

**DOI:** 10.3390/s26020612

**Published:** 2026-01-16

**Authors:** Francisco López de la Rosa, José L. Gómez-Sirvent, Roberto Sánchez-Reolid, Rafael Morales, Antonio Fernández-Caballero

**Affiliations:** 1 Insituto de Investigación en Informática de Albacete (I3A), Calle de la Investigación, 2, 02071 Albacete, Spain; francisco.lopezrosa@uclm.es (F.L.d.l.R.); jose.gomez@uclm.es (J.L.G.-S.); roberto.sanchez@uclm.es (R.S.-R.); rafael.morales@uclm.es (R.M.); 2 Departamento de Ingeniería Eléctrica, Electrónica, Automática y Comunicaciones, E.T.S. de Ingeniería Industrial de Albacete, Universidad de Castilla-La Mancha, Avenida de España, s/n, 02071 Albacete, Spain; 3 Departamento de Sistemas Informáticos, E.T.S. de Ingeniería Industrial de Albacete, Universidad de Castilla-La Mancha, Avenida de España, s/n, 02071 Albacete, Spain; 4 Departamento de Ingeniería Eléctrica, Electrónica, Automática y Comunicaciones, E.T.S. de Ingeniería Industrial de Ciudad Real, Universidad de Castilla-La Mancha, Calle Altagracia, 50, 13071 Ciudad Real, Spain

**Keywords:** data augmentation, image preprocessing, convolutional neural networks, automated inspection systems, reliability

## Abstract

Any industrial application that uses convolutional neural networks (CNNs) requires initial data and resources in order to train the models. However, the selection of models must be appropriate to the quality and quantity of the available data and computational resources. This study analyses the influence of data quantity and quality on the performance of CNN models of different complexity. Image preprocessing and image transformation data augmentation techniques are applied to generate different amounts of synthetic data with which to train the aforementioned models, shedding light on the following question: does the quality and quantity of the data or the depth of the model have more influence? Different experiments are performed using the Northeastern University (NEU) Steel Surface Defects Database, which contains surface defects found in hot-rolled steel. After analyzing the results, the authors conclude that data quality and quantity have a much greater influence than model choice. As resources and time are often limited in industry and the ultimate goal is to maximize profit by increasing efficiency, the authors encourage researchers to carefully consider the industrial application at hand and analyze the available data and resources before selecting CNN models.

## 1. Introduction

Artificial intelligence (AI) is a popular subject. New AI instruments and their applications are discussed in the news and on social media every day. The reality is that there are many AI tools that can be extremely helpful in many different aspects of people’s daily lives and work. If there is one area where AI is particularly useful and beneficial, it is probably in industry. AI can be used, for example, to forecast demand or minimize waste in a particular industry [1,2]. But the impact of AI is more noticeable in helping workers with repetitive and tedious tasks, such as visual inspection [3,4].

Traditionally, human operators have performed visual inspection tasks to ensure reliability. This involves observing a constant flow of parts to identify defects and their possible causes and consequences [5]. The visual inspection task is known to be tedious and stressful, and mistakes can be made in the classification process [6,7]. Fortunately, Industry 4.0 and its principles have irrupted into the industrial paradigm to carry out a complete revolution. The concept of Industry 4.0 is centered around the creation of smart factories. These smart factories are made up of cyber–physical systems, which are a combination of computers and physical systems that bring together the digital and physical realms [8]. This connection is made possible by networks of sensors, actuators and computers that share a constant flow of information using novel technologies such as the Internet of Things (IoT). In our case study, high resolution cameras and optical and electronic microscopes are intelligent devices that capture high-resolution images for defect identification. Thanks to machine learning (ML) and deep learning (DL), the wealth of data collected during visual inspections can now be used to automatically detect and classify defects in manufactured goods, thereby assisting workers with these tasks [9,10]. Therefore, the overall inspection chain is a CPS, in which high resolution cameras or electronic or optical microscopes act as sensors that communicate directly with a computer on which ML or DL algorithms are implemented for defect classification.

When it comes to image data and classification tasks, convolutional neural networks (CNNs) spring to mind. These are DL models composed of different layers, such as an input layer, convolutional layers, activation layers, pooling layers, fully connected layers, and output layer [11]. Each layer consists of a certain number of neurons, the weights of which are modified during network training to minimize the network’s loss. Loss is the difference between the network’s actual and expected outputs, and it is calculated according to a specific loss function. The current trend in CNN model development is to increase the depth of CNN models by adding more layers [12]. This trend has its advantages and disadvantages. While using heavy models can lead to better classification performance, training them is both resource-intensive and time-consuming [13]. Therefore, it is necessary to explore alternatives that are efficient in terms of resource consumption while maintaining the classification performance of the model.

The scientific literature agrees that CNN models require large sets of images to achieve an acceptable level of classification performance. Given the amount of data collected in each inspection process, you might think that there would be more than enough images to train these models. Fortunately, this is not the case, as the failure rate is usually very low. Therefore, it is difficult to obtain a sufficient number of images to train these models. Furthermore, not every defect is present in the same proportion. This can lead to imbalances in the dataset. There are a number of methods that can be used to solve, or at least mitigate, these problems. The main technique used to increase the amount of data available for training the model is data augmentation. This involves creating synthetic images from the original images. This can be done in a number of ways, some of which are explained in this paper. In the case of the imbalance challenge, data augmentation can be used to oversample a particular class of images. However, there are other approaches, such as downsampling (reducing the number of images of one or more classes), or a combination of oversampling and downsampling [14]. Another approach is to use evaluation metrics that account for imbalance when assessing model performance. One such metric is the F1-score [15].

Another important consideration is that industrial environments are dynamic and subject to constant change. These changes affect materials, machinery and every element of the manufacturing process. In the case of visual inspection, the characteristics of the industrial environment can result in noisy images. When the differences between classes are subtle, great detail is necessary. Noisy images can completely ruin a model’s performance [16]. This is where image preprocessing techniques can be useful. These techniques can be applied to images in a dataset to improve defect detection and generally enhance model performance, thereby increasing process reliability [17]. Some of these techniques are described in this paper.

The interest and novelty of the paper can be summarized as follows:

- The defect classification task is treated as a two-pronged challenge: (a) the data is analyzed in terms of their quantity and quality, and (b) the CNN model is studied for its classification performance and resource consumption.
- This research involves analyzing the impact of data augmentation and image preprocessing on the classification performance of three different models: a basic CNN model, a lightweight CNN model, and a deep CNN model.
- The research is supported by benchmark data from the NEU Surface Defect Database [18], an open-source dataset containing images of six typical surface defects that occur in the hot rolling process of steel plates.
- The results emphasize the importance of data for image classification problems using CNN models. Through this study, the authors aim to demonstrate that, provided the data is of an adequate quality and quantity, a simple CNN model can rival a highly sophisticated model in terms of accuracy while consuming much fewer resources.

The rest of the work is organized as follows. Section 2 contains (i) a description of the role of sensors in visually inspecting surface defects, (ii) information regarding the NEU dataset and a description of the different types of defects it contains, (iii) a brief explanation of image preprocessing techniques and their use in this study, and (iv) a shallow dive into data augmentation and its application in this work. Section 3 presents the three different models (basic CNN, lightweight CNN, and deep CNN) used to carry out this research. Section 4 describes the methodology used to train the previously presented models. Section 5 includes the experimental results achieved by the different models in terms of both classification performance and resource consumption. Finally, Section 7 collects conclusions and suggestions for future work.

## 2. Background

Section 2 is divided into four subsections. The first subsection discusses the role of sensors in surface defect visual inspection and data collection. The second subsection provides a detailed overview of the NEU Dataset, which was used to conduct the experiments in this study. The third subsection presents the image preprocessing techniques employed in this research. The final subsection introduces data augmentation techniques and their application in this work.

### 2.1. The Role of Sensors in Surface Defect Visual Inspection

Sensors play a very important role in visual inspection tasks for identifying surface defects [19]. Regardless of the classification method used, whether traditional manual or innovative automatic, the process involves analyzing images of the material’s surface defects. In our case study, the images must be of a high resolution, since the defects involved in the hot rolling of steel are usually very small. Due to their small size, the defects must be located beforehand in order to take the high-resolution images that will later be analyzed by experts or ML/DL algorithms.

**Figure 1:**
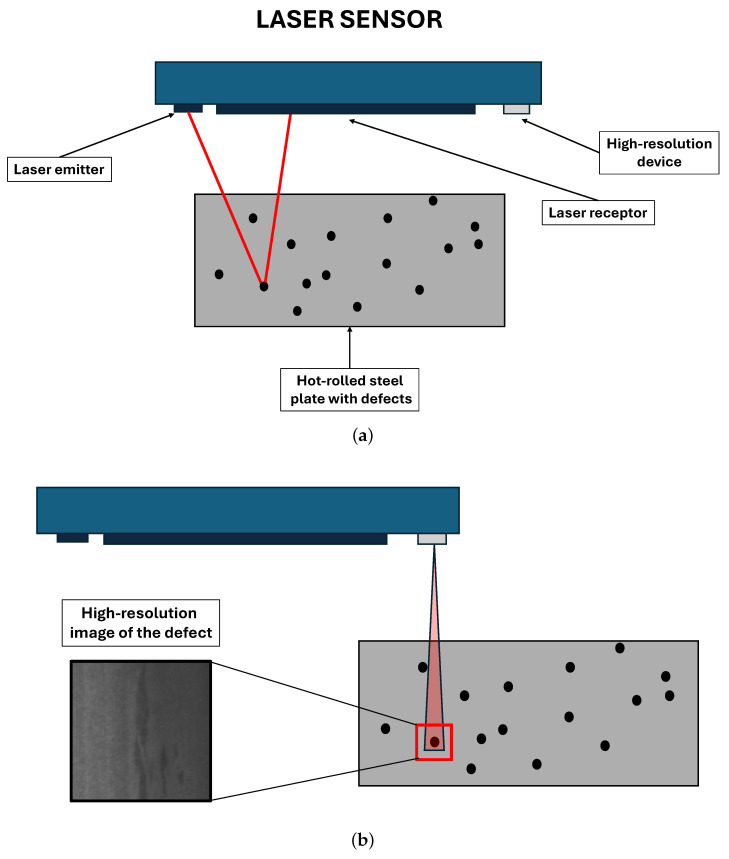
Sensors used in the visual inspection of surface defects on hot-rolled steel plates. (**a**) Laser sensor for defect location. (**b**) High-resolution image acquisition.

This is where sensors come into play. Specifically, optical sensors. A typical setup for visually inspecting small surface defects uses laser sensors. The defect detection process begins with a laser scanning a specific area of the material’s surface. In this case, the material is hot-rolled steel. The sensor receives the reflection of the projected beam. When the laser beam hits a defect, the reflection is different to what is expected. When an anomaly is detected, its coordinates are sent to a high-resolution device. Depending on the size of the defect, this device may be a high-resolution camera or a microscope, which is used to capture an image of the defect. Then, the high-resolution image of the defect is sent to experts for classification, or to data scientists for use in building datasets to train DL or ML models. Figure 1 aims to illustrate this process.

### 2.2. NEU Dataset

**Figure 2:**
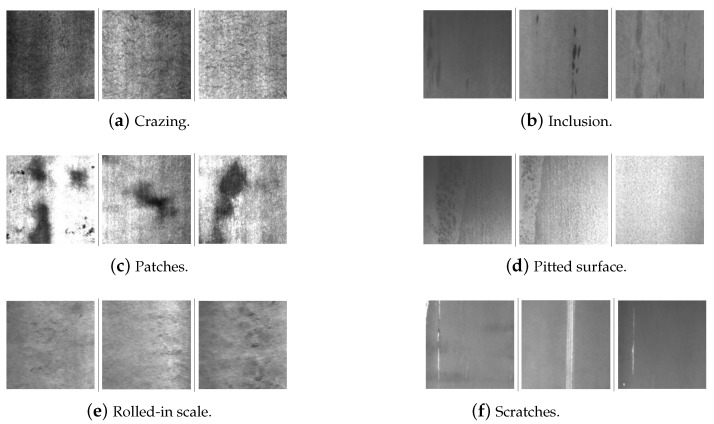
NEU database defects.

The NEU database contains information on surface defects that typically occur during the hot rolling process of steel plates. The NEU dataset contains six different classes of surface defect: *crazing*, *inclusion*, *patches*, *pitted surface*, *rolled-in scale*, and *scratches*. The NEU dataset contains 300 grayscale images of each class, for a total of 1800 images. Therefore, the imbalance problem does not affect the NEU dataset because each class contains the same number of images. More specifically, the size of each image in the dataset is 200×200 pixels. Figure 2 illustrates some representative samples of defects for each class. Two peculiarities of the dataset are worth mentioning: (i) there is a high variability within defects of the same class, probably caused by different lighting, some noise, changes in the own steel plate, etc., and (ii) there is a certain similarity between some of the classes in the dataset (i.e., *patches*, *pitted surface*, and *inclusion*).

In addition, the main characteristics of each of the six classes of defects collected in the NEU database are outlined in Table 1.

Finally, it is worth mentioning that this dataset has been used in countless articles as the main database for defect detection [20,21] or classification [22,23,24], which is one of the main reasons why it has been used.

### 2.3. Image Preprocessing

**Figure 3:**
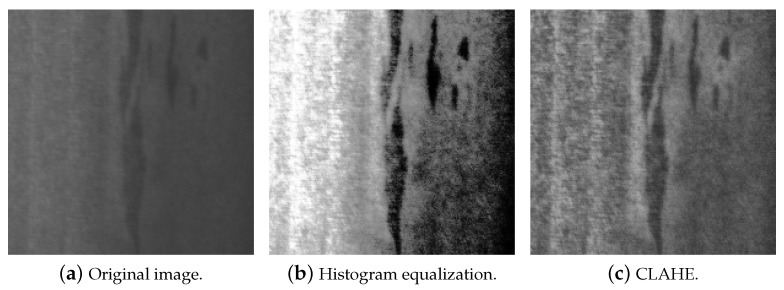
Histogram equalization vs. CLAHE.

Data quality is a key consideration in defect classification tasks. Enhancing the contrast between defects and their background in images is critical to improving the performance of classification models [25]. Image enhancement can be achieved using image preprocessing techniques. In this study, the authors focus on histogram equalization techniques [26], which improve the contrast in an image. The process involves calculating the histogram of an image’s intensity values and then distributing these values evenly across the intensity range to enhance contrast. In cases where the pixel intensity values of the defect or background are highly heterogeneous, a modification of histogram equalization called adaptive histogram equalization (AHE) is worth using [27]. AHE splits the image into different regions and performs histogram equalization on each of the regions. However, using AHE can lead to a huge increase in noise in the image. This is where contrast limited adaptive histogram equalization (CLAHE) [27] comes in. CLAHE performs the same operation as AHE, with one major difference: the histogram used to perform AHE is clipped at a certain defined value to avoid noise amplification. Figure 3 shows the result of applying simple histogram equalization and CLAHE to a sample of the dataset.

### 2.4. Data Augmentation

Data augmentation is the main technique used to generate synthetic data when there is insufficient data to properly train a particular DL or ML model [28]. As mentioned above, CNN models require large sets of images to perform optimally in classification tasks. Therefore, data augmentation is essential for most work involving CNNs for image classification. Data augmentation techniques can be divided into two main groups: DL-based and image transformation-based [29]:

- *DL-based data augmentation techniques* use DL models to generate synthetic data. The most relevant DL models for this purpose are probably generative adversarial networks (GANs) [30]. These models consist of two modules: a generator and a discriminator. The generator learns features from the original data and uses them to produce synthetic data. Meanwhile, the discriminator evaluates the synthetic images by comparing them with the original images, rejecting any that do not match. In other words, the generator’s aim is to produce images that are so similar to the original data that the discriminator cannot distinguish between them. There are many examples of work using GANs for image augmentation in defect classification tasks [31,32,33]. Another DL tool used to generate synthetic data is neural style transfer (NST) [34]. NST involves using CNNs to transfer the style of one image to another without altering its content. While this approach is interesting from an artistic perspective, there is currently no evidence to suggest that NST can be used for defect classification tasks.
- Data augmentation techniques based on *image transformation techniques* create new images by modifying the original images. Some image transformation techniques are the application of kernels or filters (Gaussian, median, mean), random erasure of parts of the original images, color space transformations, or geometric transformations (flipping, rotating, scaling, and/or shifting the original images). In fact, image transformation-based data augmentation techniques are less complex and resource-intensive than DL approaches. Moreover, it is still difficult to assess the quality and performance of DL approaches [35].

**Figure 4:**
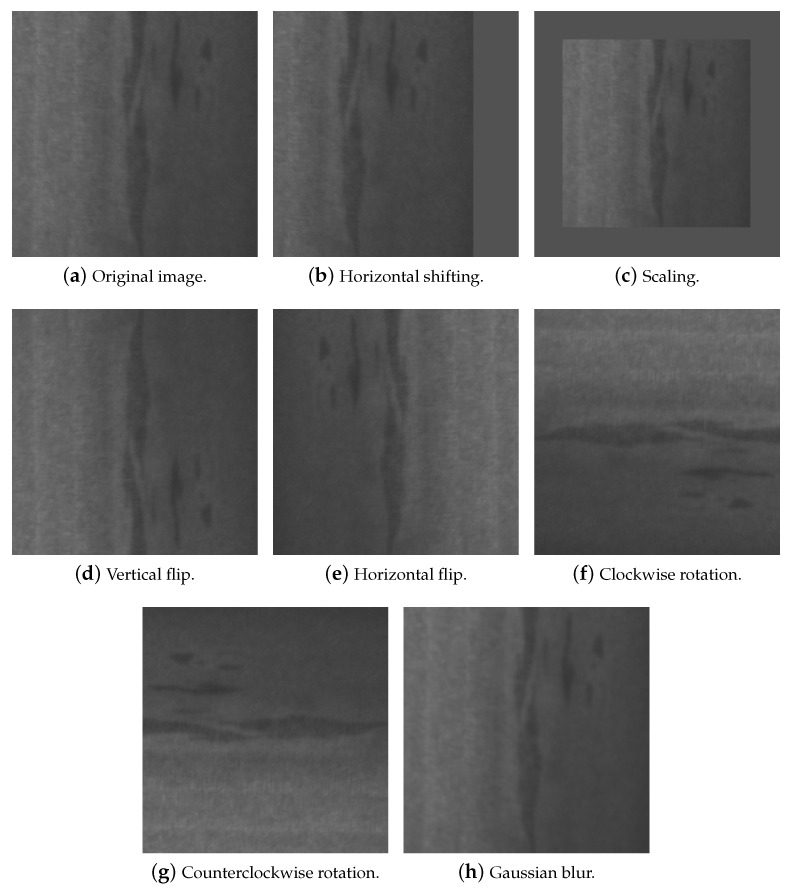
Data augmentation techniques employed in this work.

As this study aims to analyze the performance of CNN models for different data volumes and image preprocessing, DL-based data augmentation techniques are not investigated, as they are more complex and resource-intensive, and the results are difficult to interpret and quantify. The main focus of this research is therefore image transformation-based data augmentation techniques. A total of five different operations are used. Figure 4 illustrates the output of each data augmentation technique applied to an original image from the NEU database. These techniques are as follows:

- *Horizontal shifting* randomly shifts pixels to introduce horizontal variations in their position. In this study, the maximum displacement is limited to 20% of the width of the image. If necessary, the image is filled with the average pixel value of the original image to maintain the original dimensions (see Figure 4b).
- *Scaling* applies a random scaling factor between 0.5 and 1.5, proportionally changing the dimensions of the image. If necessary, the synthetic image will be filled with the average pixel value of the original image to maintain the original dimensions (see Figure 4c).
- *Flipping* (horizontal and vertical) flips the image horizontally (see Figure 4e) or vertically (see Figure 4d).
- *Rotation* (clockwise and counterclockwise) consists of rotating an image by a certain number of degrees around a given point, which is usually the center. Rotation can be clockwise (see Figure 4f) or counterclockwise (see Figure 4g). In this work, counterclockwise rotation is performed by rotating the image −90° and clockwise rotation by rotating the image 90°.
- *Gaussian blur* smooths the image by reducing the contrast between adjacent pixels (see Figure 4h).

The combination of these techniques and image preprocessing will then be used to generate different datasets that will be used to train the models presented below for future comparison. To ensure experimental reproducibility, the authors would like to point out that the random seed used for all data argumentation transformations is 42.

## 3. The Three CNN Models

This section introduces the three models to be compared in this study. First, the basic CNN architecture is presented. Next, we introduce the lightweight model. Finally, the deep CNN model is described.

### 3.1. The Basic CNN

The idea is to build a very simple CNN model, train it, and then compare its results with those of more complex models. The proposed basic model architecture is shown in Table 2. The basic model combines five different convolutional blocks. Each convolutional block comprises a convolutional layer, a max-pooling layer, and ReLU activation [36]. The output of the fifth convolutional block is flattened by a flatten layer. The output then goes through three dense layers. The last layer is activated by the SoftMax function [37].

### 3.2. The Lightweight CNN

The second model investigated in this paper is an example of a lightweight CNN model. In the search for the most subtle data pattern recognition, CNNs are becoming increasingly deep with millions of trainable parameters [38]. While this is not a fundamental problem, it is true that there are constraints such as training time and available resources. Additionally, some applications may require models that can be embedded in everyday devices, such as smartphones, and perform classification in a few milliseconds [39].

Specifically, the lightweight model used for conducting part of the experiments in this research is SqueezeNet [40], which was designed to combine good classification performance with efficiency in terms of resource consumption. This is achieved by implementing the fire module. The fire module is the concatenation of a *squeeze* layer consisting of 1 × 1 kernels feeding an *expand* layer combining 1 × 1 and 3 × 3 kernels. ReLU activation is introduced before and after the *expand* layer. The fire modules have three different parameters that can be tuned:

- $s_{1\times 1}$: number of 1 × 1 kernels in the *squeeze* layer.
- $e_{1\times 1}$: number of 1 × 1 kernels in the *expand* layer.
- $e_{3\times 3}$: number of 3 × 3 kernels in the *expand* layer.

**Figure 5:**
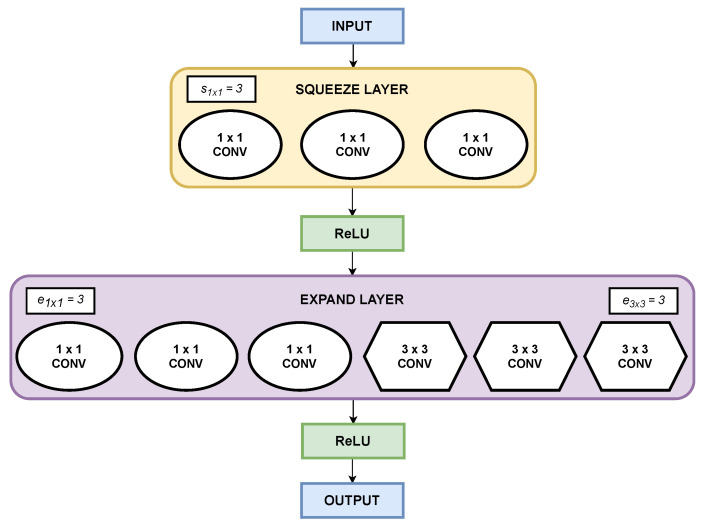
Fire module.

To minimize the number of input channels, $s_{1\times 1}$ is constrained to be less than the sum of $e_{1\times 1}$ and $e_{3\times 3}$. Therefore, the fire module introduces a huge parameter reduction by replacing 3 × 3 kernels with 1 × 1 kernels and reducing the number of input channels to the kernels. Figure 5 shows the fire module with all its elements. It should be noted that, although there are several SqueezeNet architectures, the authors decided to implement the simplest model due to its lightweight and low-complexity nature. The architecture of the selected SqueezeNet CNN model can be found in Table 3.

### 3.3. The Deep CNN

Finally, the deep CNN model chosen was ResNet50 [41]. ResNet50 is a well-known residual model that has shown great performance in similar defect classification tasks [42]. Although ResNets are considered deep CNNs, they were designed to mitigate the degradation issue that many other deep CNN models present. Once the training curves of deep CNN models begin to converge, their accuracy becomes saturated and then drops rapidly. Far from solving the degradation problem, adding new layers makes it worse, not better. Thus, ResNets are not the typical deep CNN.

**Figure 6:**
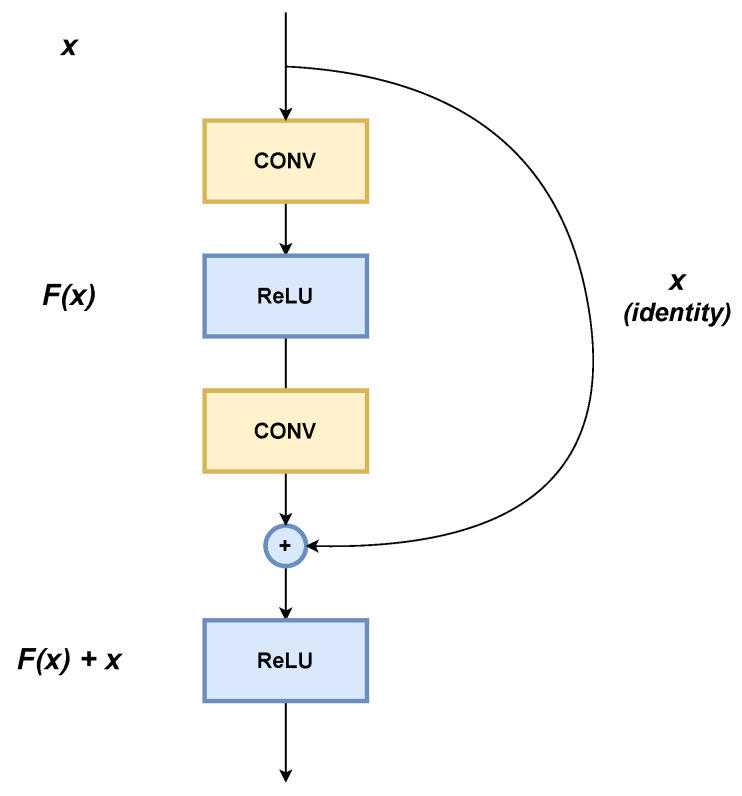
Residual block.

The approach taken by ResNets to overcome the degradation problem is the use of residual blocks (RBs). RBs are structures in which one layer is connected to the next, and to the one, two, or three layers ahead, by a kind of shortcut, so that the neurons in one layer learn only a residual correction from the weights of the neurons in the previous layer, rather than changing all their weights. Figure 6 is intended to illustrate how RBs work, in which F(x) is the output of the system after convolutions, *x* represents the function to be fitted, and ReLU denotes the activation function implemented in this block [41].

Of the various ResNet models available, the authors have chosen ResNet50 based on previous experience with this model [42]. The architecture of ResNet50 can be found in Table 4.

## 4. Methodology

**Figure 7:**
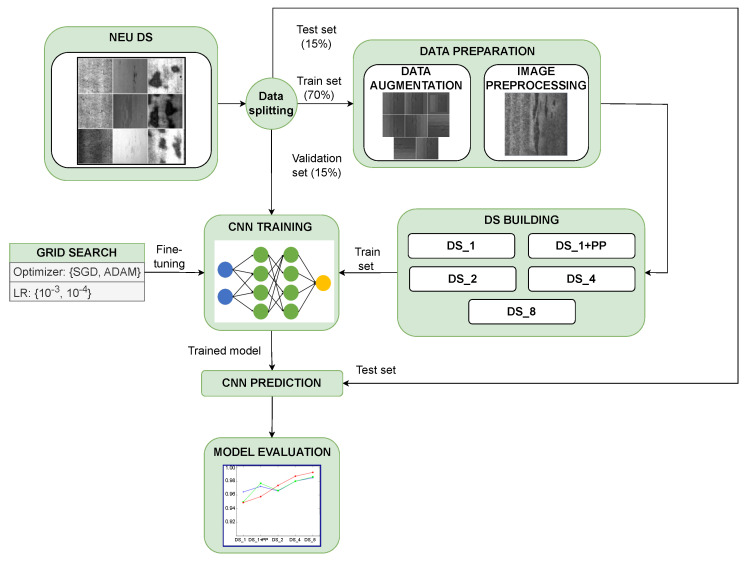
Proposed methodology for training the CNN models.

This section describes the proposed methodology for training the presented models, which is illustrated in Figure 7. First, the process of splitting the data and building the different datasets is explained. Next, the hyperparameter tuning strategy for the models is described. Finally, the evaluation metrics used to assess the classification performance of each model are presented.

### 4.1. Data Preparation

Five different datasets were created using a combination of image preprocessing and data augmentation. *DS_1* is the original NEU dataset. *DS_1+PP* is the original NEU dataset with its images preprocessed using the CLAHE algorithm. *DS_2* is the first augmented dataset. Its training set contains twice as many images as the previous one. Finally, *DS_4* and *DS_8* datasets have training sets with four and eight times the number of images, respectively, of the original training set. All images in the augmented datasets are also preprocessed with CLAHE.

**Figure 8:**
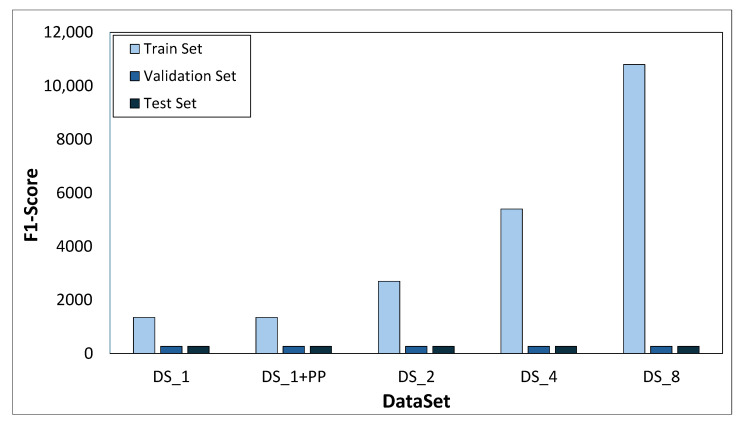
Datasets.

Each model is trained 100 times, and the results presented are the average of the 100 iterations. The training, validation, and test sets are randomly sampled from the original images in each of these 100 iterations. The proportions of the different batches are as follows: 70% training set, 15% validation set, and 15% test set. After splitting, the different datasets are built. Therefore, one, three, or seven synthetic images are generated from each image of the training set using data augmentation techniques, in order to build the different augmented datasets (*DS_2*, *DS_4* and *DS_8*). CLAHE is also used when necessary. Therefore, the idea is to randomize the data flow to build the different datasets to ensure the validation of the proposed methodology. The number of images in each of the created datasets is depicted in Figure 8.

### 4.2. Hyperparameter Tuning

Each CNN has a number of design parameters that must be configured to optimize learning. These design parameters are called hyperparameters, and their tuning is a key factor in achieving good classification performance from a CNN model [43]. There are several strategies that can be used to fine-tune the hyperparameters. Despite its low efficiency and being considered a brute force algorithm, grid search is perfect for performing a search from a small set of hyperparameters [44]. In this work, since there are only a few hyperparameters to be tuned, the authors have chosen the grid search algorithm to find the optimal hyperparameter configuration of each model. Grid search tests every possible hyperparameter combination and ensures that the best hyperparameter tuple is found, which is always determined using a certain performance metric. The following hyperparameters are considered for fine-tuning the model:

- *Learning rate* (LR) determines the rate at which the weights of the model’s neurons are updated as a function of the model’s error in the previous step. The LR value is usually between 0 and 1. Low values can lead to training errors, such as not finding the global minimum of the error function, while high values lead to training instability. Therefore, it is important to find the optimal LR value to improve the performance of the model. As a note, the error of a CNN model is understood as the difference between the actual output of the model and the expected output of the model, and is calculated using a specific loss function. In multi-class classification scenarios, categorical cross entropy (CCE) is the most commonly used loss function.
- *Optimizer* aims to minimize the loss computed by the above loss function (CCE in this case) by managing the learning rate to modify the weights of the neurons in each layer. There are several optimizers available for training a CNN model. Some of them are Adadelta, Adagrad, Adamax, or RMSprop. However, if there are two optimizers that stand out from the rest by their popularity and versatility, it would be adaptive moment estimation (ADAM) and stochastic gradient descent (SGD) [45] and stochastic gradient descent (SGD) [46]. These two optimizers have been included in the grid search.

### 4.3. Model Evaluation

The test set is used to evaluate the performance of the models trained for each hyperparameter combination of the grid search algorithm. The metric chosen for this evaluation is the F1-score. The F1-score is defined as the harmonic mean of precision and recall, whose equations and definitions are presented below:

- *Precision* (P) refers to the proportion of successful positive predictions. It is determined in terms of true positives (TP) and false positives (FP), as follows:
  (1) $$P=\frac{TP}{TP+FP}$$
- *Recall* (R) presents the proportion of relevant predictions; it is computed in terms of TP and false negatives (FN), as follows:
  (2) $$R=\frac{TP}{TP+FN}$$
- *F1-score* (F1) is the harmonic mean between P and R. It is calculated as
  (3) $$F1=\frac{2\cdot P\cdot R}{P+R}\cdot 100$$

The optimal model is the one trained with the combination of hyperparameters that gives the highest F1-score and the lowest validation loss. The authors would like to mention that if the aim of the study were to be to optimize the classification performance of a specific model for a particular task, more metrics such as confusion matrices would have been included. Furthermore, the classification performance of the models for each class of the dataset would have been analyzed. However, this study aims to compare the classification performance of models of different complexities when dealing with different quantities and qualities of data. For this reason, the authors have decided to use a single metric for comparison. The F1-score was chosen as it is the most commonly used metric for classification problems.

## 5. Results

This section collects the system specifications used to perform the various experiments, along with the results obtained in each of these experiments.

### 5.1. Training Specifications

Each experiment was performed on a workstation computer with the following hardware specifications: 3.80 GHz Intel i7-10700KF v8 CPU, 32 GB RAM, and NVIDIA GeForce RTX 2070 SUPER 8 GB. The Keras [47] and TensorFlow [48] libraries were used to train the models, with Pycharm [49] as the integrated development environment (IDE) and Python 3.8 [50] as the programming language.

### 5.2. Results of the Grid Search

As introduced above, grid search was chosen as the hyperparameter optimization algorithm. The hyperparameters to be tuned are the LR and the optimizer. Table 5 lists the different values for the LR and the optimizer that were used in the experiments. Moreover, the results for every hyperparameter configuration for each model and dataset are shown in Table 6. Looking closely, it seems clear that the best configuration of hyperparameters is the one with ADAM as the optimizer and an LR of $10^{-4}$. In fact, this configuration provides the best classification performance for all models and datasets.

### 5.3. Analysis of the Results

**Figure 9:**
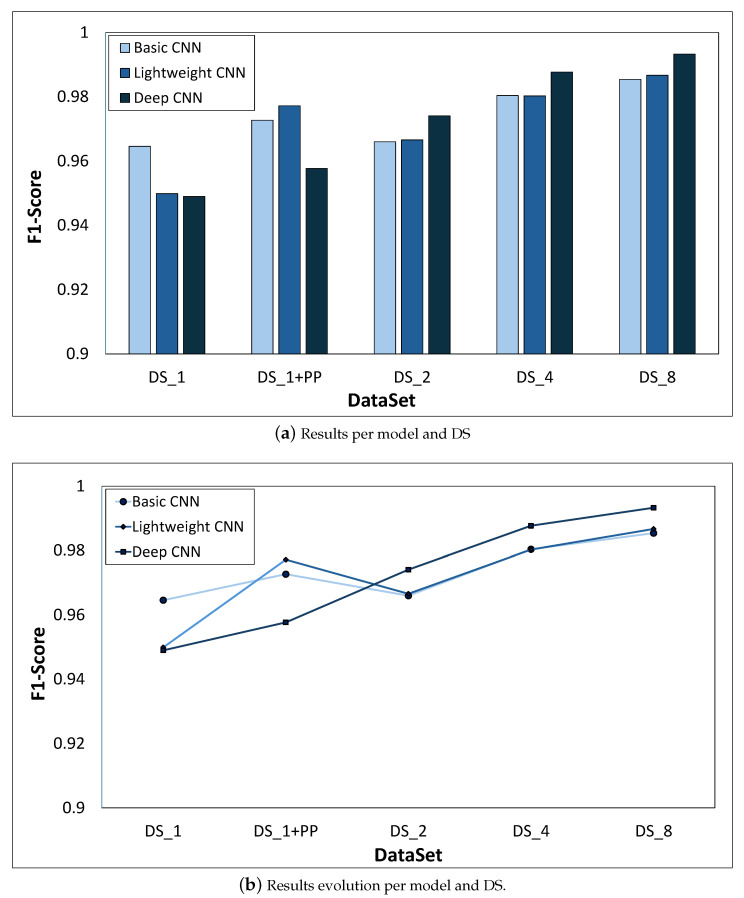
Results of the grid search for all datasets.

The results obtained in this work for each dataset are analyzed here after the selection of the best hyperparameter tuple. The results for the different DS and models are shown in Figure 9a.

- **DS_1.** The basic CNN model (0.9646 F1-score) is the one that performs better when each model is trained with the original DS, closely followed by the lightweight model (0.9499 F1-score), while the deep CNN (0.9490 F1-score) is the worst performing model in this scenario. However, the difference between the performance of the models is really small.
- **DS_1+PP.** In this case, the lightweight model (0.9772 F1-score) is the best performing model, followed by the basic CNN model (0.9727 F1-score). Once again, the deep CNN (0.9577 F1-score) is the worst performing model. Again, the performance of the models is very similar.
- **DS_2.** The best performing model is the deep CNN (0.9741 F1-score), followed by the lightweight model (0.9666 F1-score). In this scenario, the worst performing model is the basic CNN (0.9660 F1-score). The comparison shows that there is not much difference between the performance of the three models.
- **DS_4.** In this scenario, the model that outperforms the rest is the deep CNN (0.9877 F1-score). The next model in terms of classification performance is the basic model (0.9804 F1-score). Finally, the worst performing model is the lightweight CNN (0.9803 F1-score). Again, the comparison shows that there is not much difference between the performance of the three models.
- **DS_8.** Here, the best performing model is the deep CNN (0.9933 F1-score), followed by the lightweight model (0.9867 F1-score). In this scenario, the worst performing model is the basic CNN (0.9854 F1-score). As in the other scenarios, the comparison shows that there is not much difference between the performance of the three models.

The results suggest that there are no big differences whether one model or another is chosen. Figure 9b shows that the performance difference between models is really small. In fact, the difference between the best and worst models for the largest dataset is less than 0.8%. The curves for the light and basic CNN models almost overlap. As for the deep CNN, it can be observed that it performs worse for the first two datasets (DS_1 and DS_1+PP), while it starts to outperform the rest when data augmentation is applied.

The other relevant aspect that can be highlighted after analyzing the figure is the influence of data augmentation and image preprocessing on the classification performance of the models. From DS_1 to DS_1+PP (from original to preprocessed dataset), there is an improvement in the performance of each model. The performance of the basic CNN model improves by 0.81%, the lightweight model by 2.73%, and the deep CNN model by 0.87%. In terms of data augmentation, the influence on the classification performance is much more noticeable. The difference between the F1-scores obtained by the models when trained with DS_1 (the original DS) and DS_8, the largest DS, is 2.08%, 3.68%, and 4.43% for the basic, lightweight, and deep CNN models, respectively. Therefore, in this application, the influence of data quantity and quality is more important than the model.

### 5.4. Training Time and Number of Trainable Parameters

Since the performance difference is almost non-existent, it is time to weight other variables such as the training time and the number of trainable parameters of each model. Table 7 collects the approximate average training time per epoch and the number of trainable parameters for each model. The authors prefer to use these metrics to evaluate the computational cost of these models because the total time required to train each model would change completely depending on the training conditions used (e.g., maximum number of epochs, early stopping conditions, etc.).

From the table, two groups of models can be clearly distinguished. On the one hand, both the lightweight model and the basic model have identical average times per epoch (7 s) and a reduced number of trainable parameters (less than one million). On the other hand, the deep CNN model has an average time per epoch of 73 s and more than 23.5 million trainable parameters. Since the trainable parameters of a model are closely related to the size and training time of the model, and certainly to the resource consumption, the deep CNN model is significantly more resource intensive than the other two models.

Thus, after analyzing the different results obtained, it can be concluded that it is more interesting to carry out good data processing, improving the quality and quantity of the data through the use of preprocessing and data augmentation, than to choose a deep CNN model and leave the data processing aside. However, since the classification performance of the three models studied is very similar (slightly better in the case of the deep CNN model), the researcher can choose one or another model depending on the resources available and the nature of the industrial application.

Therefore, it is important to perform a thorough analysis of the particular industrial application and the available data and resources before selecting a model. In fact, the limiting factor in achieving high classification performance is usually the data, not the model.

### 5.5. Comparison with Recent Relevant Work

This section provides a brief comparison of the results of this study with those of similar studies that used the NEU dataset. Table 8 shows this comparison.

The table clearly shows that the models trained in this study perform at least as well as the current best classification models. Please note that the studies selected for comparison are of a different nature. In [22], the authors tested both deep CNN models (e.g., VGG, ResNet) and lightweight models (e.g., YOLO, MobileNet) to classify the defects in the NEU dataset. In [23], the authors developed a CNN from scratch to classify the defects in the NEU database. Finally, the authors in [24] presented another combination of deep CNN (e.g., GoogleNet, ResNet) and lightweight models (e.g., MobileNetV2) to classify the defects in the NEU database. Therefore, the works shown in the table include CNNs that have been designed from scratch, as well as deep and lightweight CNN models. This makes the comparison fair, as it is consistent with our own research.

## 6. Strengths and Limitations

Like all studies, this one has its strengths and limitations. Its greatest strength is probably the message it sends that resources should be invested in improving the quantity and quality of data, rather than developing increasingly complex CNN models. The main limitation is that the conclusions can only be extrapolated to the NEU dataset and similar ones. It remains to be seen what would happen with much more complex datasets. Another limitation is that only three models were tested, although these are quite representative.

## 7. Conclusions

This paper presents the results of a study investigating the impact of data augmentation and image preprocessing on the classification performance of CNN models using a dataset of surface defects on hot-rolled steel. To this end, various datasets were created using image preprocessing and data augmentation techniques: DS_1 (the original DS), DS_1+PP (the original DS once preprocessed with the CLAHE algorithm), DS_2 (with its training set containing twice the number of images of DS_1), DS_4 (with its training set containing four times the number of images of DS_1), and DS_8 (with its training set containing eight times the number of images of DS_1).

The previously generated datasets were then used to train CNN models with different characteristics: a basic CNN model designed from scratch, a lightweight CNN model (SqueezeNet), and a deep CNN model (ResNet50). Each of these models was fine-tuned using a grid search algorithm that played with two main hyperparameters: learning rate and optimizer. The idea was to train each of these models on each dataset to analyze their performance. The results showed that despite their different characteristics (the basic and lightweight CNN models are much less time- and resource-consuming than the deep CNN model), all three models performed similarly for each dataset. This demonstrates that, for this application, the influence of data augmentation and image preprocessing is more relevant than the model itself.

In this study, the authors found that data is more important than the model when working with the NEU database. It is believed that these results can be extrapolated to other works involving datasets of a similar complexity. In these cases, since the limiting factor in achieving high classification performance is usually the data and not the model, a thorough analysis of the industrial application and the available data and resources must be carried out before selecting a model, with a view to increasing efficiency and maximizing profit while maintaining acceptable reliability. Although this contribution is aimed at the industrial sector, the main conclusion of this work may be useful in many other fields. The general trend is to use highly sophisticated CNN models to identify subtle patterns when classifying data. This work demonstrates how shortcuts can be taken by making effective use of available data.

In terms of future work, one possible research direction would be to apply the same methodology to the Xsteel Surface Defect Dataset [51], which is a similar database to the one used in the present study and offers new challenges. Another research line could involve the exploration of the field of GANs as a tool for data augmentation. A comparative study could even be performed to evaluate the quality of data generated by GANs and geometric transformations in an application such as the one presented in this paper. In the same line, other data augmentation approaches based on consistency modeling, like the DefectDiffu approach [52], can also be studied for this purpose. Another research line could involve testing powerful, lightweight, on-the-fly models such as YOLOv7 [53], or DL alternatives to CNNs, such as powerful transformers (e.g., Pyramid Vision Transformer [54]). In parallel research, we are still making progress in the detection of unknowns. However, this research is still at an early stage.

## Figures and Tables

**Table 1:** Description of the defect classes.

Defect Class	Description
*Crazing*	This class of defects takes the form of cracks on the plate’s surface (see Figure 2a).
*Inclusion*	The defects in this category are caused by impurities being pressed against the steel plate during the hot rolling process (see Figure 2b).
*Patches*	The defects in this class take the form of multiple irregularly shaped spots over the entire surface of the steel (see Figure 2c).
*Pitted surface*	The defects in this class take the form of small circular patterns that penetrate the metal. These patterns are usually caused by corrosion (see Figure 2d).
*Rolled-in scale*	The defects included in this class are irregular signs of wear caused by the mill scale introduced into the steel during rolling (see Figure 2e).
*Scratches*	The defects included in this class are linear marks left on the material by the rolls themselves during the hot rolling process (see Figure 2f).

**Table 2:** Specification of the basic CNN architecture.

Layer	Output Size	Filter Size
*Input*	200 × 200 × 1	
*Conv.* 1	196 × 196 × 32	5 × 5
*MaxPool* 1	98 × 98 × 32	2 × 2
*Conv.* 2	94 × 94 × 32	5 × 5
*MaxPool* 2	47 × 47 × 32	2 × 2
*Conv.* 3	44 × 44 × 64	4 × 4
*MaxPool* 3	22 × 22 × 64	2 × 2
*Conv.* 4	19 × 19 × 64	4 × 4
*MaxPool* 4	9 × 9 × 64	2 × 2
*Conv.* 5	8 × 8 × 128	2 × 2
*MaxPool* 5	4 × 4 × 128	2 × 2
*Flatten* 1	2048	
*Dense* 1	512	
*Dense* 2	256	
*Dense* 3	6	

**Table 3:** Specification of the lightweight (SqueezeNet) CNN architecture.

Layer	Output Size	Filter Size/Stride	$s_{1\times 1}$	$e_{1\times 1}$	$e_{3\times 3}$
*Input*	200 × 200 × 1				
*Conv. 1*	200 × 200 × 32	7 × 7/(×32)			
*MaxPool 1*	100 × 100 × 32	2 × 2/2			
*Fire 2*	100 × 100 × 32		8	16	16
*Fire 3*	100 × 100 × 32		8	16	16
*Fire 4*	100 × 100 × 64		16	32	32
*MaxPool 4*	50 × 50 × 64	2 × 2/2			
*Fire 5*	50 × 50 × 64		16	32	32
*Fire 6*	50 × 50 × 128		32	64	64
*Fire 7*	50 × 50 × 128		32	64	64
*Fire 8*	50 × 50 × 256		64	128	128
*MaxPool 8*	25 × 25 × 256	2 × 2/2			
*Fire 9*	25 × 25 × 256		64	128	128
*Conv. 10*	25 × 25 × 6	1 × 1/1(×6)			
*AveragePool 10*	1 × 1 × 6				

**Table 4:** Specification of the deep (ResNet50) CNN architecture.

Layer	Output Size	Filter Size/Stride	
*Input*	200 × 200	-	
*Conv. 1*	112 × 112	7 × 7 × 64/2	
*Conv. 2*	56 × 56	3 × 3 max pool/2	
		1 × 1 × 64	
		3 × 3 × 64	×3
		1 × 1 × 256	
*Conv. 3*	28 × 28	1 × 1 × 128	
		3 × 3 × 128	×4
		1 × 1 × 512	
*Conv. 4*	14 × 14	1 × 1 × 256	
		3 × 3 × 256	×6
		1 × 1 × 1024	
*Conv. 5*	7 × 7	1 × 1 × 512	
		3 × 3 × 512	×3
		1 × 1 × 2048	
	1 × 1	average pool, 1000-d fc, softmax	

**Table 5:** Grid search hyperparameters considered for tuning of the CNN.

Hyperparameters	Values
*Optimizer *	ADAM, SGD
*LR*	$10^{-3}$, $10^{-4}$

**Table 6:** Grid search results for the different CNN models.

			DS_1		DS_1+PP		DS_2		DS_4		DS_8	
CNN	Opt.	LR	F1	Loss	F1	Loss	F1	Loss	F1	Loss	F1	Loss
**Basic**	ADAM	$10^{-3}$	0.6626	0.7233	0.9695	0.1086	0.9586	0.1149	0.9719	0.0686	0.9754	**0.0510**
		$10^{-4}$	**0.9646**	**0.0858**	**0.9727**	**0.0558**	**0.9660**	**0.0995**	**0.9804**	**0.0513**	**0.9854**	0.0628
	SGD	$10^{-3}$	0.9362	0.2222	0.9300	0.2001	0.9551	0.1548	0.9657	0.1140	0.9753	0.0878
		$10^{-4}$	0.8284	0.5482	0.9083	0.2621	0.9124	0.2308	0.9409	0.2217	0.9536	0.1284
**Light**	ADAM	$10^{-3}$	0.4404	1.1108	0.8809	0.3291	0.7843	0.4912	0.7957	0.4321	0.8833	0.2571
		$10^{-4}$	**0.9499**	**0.1672**	**0.9772**	**0.1341**	**0.9666**	**0.1379**	**0.9803**	**0.1058**	**0.9867**	**0.0777**
	SGD	$10^{-3}$	0.0476	1.8681	0.6566	0.9588	0.9208	0.3444	0.9648	0.2338	0.9750	0.1542
		$10^{-4}$	0.0476	1.8897	0.0476	1.8898	0.0476	1.8907	0.1256	1.8762	0.0660	1.8294
**Deep**	ADAM	$10^{-3}$	0.8264	0.6860	0.8594	0.5544	0.9604	0.1255	0.9830	0.0380	0.9919	0.0067
		$10^{-4}$	**0.9490**	**0.2100**	**0.9577**	**0.1441**	**0.9741**	**0.0480**	**0.9877**	**0.0163**	**0.9933**	**0.0050**
	SGD	$10^{-3}$	0.8883	0.4318	0.9165	0.3261	0.9575	0.1430	0.9726	0.1014	0.9809	0.0832
		$10^{-4}$	0.6187	1.0451	0.7034	0.8820	0.8820	0.3569	0.9405	0.1660	0.9649	0.0912

**Table 7:** Average time per epoch and trainable parameters.

Model	Avg. Time per Epoch (s)	Trainable Parameters
Basic CNN	7	816,998
Lightweight CNN	7	254,710
Deep CNN	73	23,540,614

**Table 8:** Comparison with related works.

Reference	CNN Model	F1-Score	Accuracy
	VGG-16	0.884	—
	VGG-19	0.894	—
	ResNet50	0.920	—
[22]	DSTEELNet	0.956	—
	MobileNet	0.920	—
	Yolov5	0.836	—
	Yolov5-SE	0.886	—
	CNN own design	—	0.991
[23]	CNN own design no augmentation	—	0.883
	GoogleNet	—	0.972
	ResNet18	—	0.981
[24]	CNN-T	—	0.989
	MobileNetV2	—	0.986
	VIT	—	0.961
	**Simple CNN **	**0.985**	**-**
**Ours**	**SqueezeNet**	**0.987**	**-**
	**ResNet50**	**0.993**	**-**

## Data Availability

The original contributions presented in this study are included in the article. Further inquiries can be directed to the corresponding author.

## Abbreviations

The following abbreviations are used in this manuscript:

AHE	Adaptive histogram equalization
AI	Artificial intelligence
CCE	Categorical cross entropy
CNN	Convolutional neural network
CLAHE	Contrast limited adaptive histogram equalization
CPS	Cyber–physical system
DL	Deep learning
DS	Dataset
F1	F1-score
FN	False negatives
FP	False positives
GAN	Generative adversarial network
IoT	Internet of Things
LR	Learning rate
ML	Machine learning
NEU	Northeastern University
NST	Neural style transfer
P	Precision
R	Recall
RB	Residual block
TP	True positives
